# Evaluation of the Eazyplex^®^ *Candida* ID LAMP Assay for the Rapid Diagnosis of Positive Blood Cultures

**DOI:** 10.3390/diagnostics14192125

**Published:** 2024-09-25

**Authors:** Arvid Berlau, Sylvia Stoll, Birgit Edel, Bettina Löffler, Jürgen Rödel

**Affiliations:** Institute of Medical Microbiology, Jena University Hospital, Friedrich Schiller University, 07747 Jena, Germany; arvid.berlau@med.uni-jena.de (A.B.); sylvia.stoll@med.uni-jena.de (S.S.); birgit.edel@med.uni-jena.de (B.E.); bettina.loeffler@med.uni-jena.de (B.L.)

**Keywords:** *Candida*, sepsis, blood culture, rapid diagnostic test, LAMP

## Abstract

Rapid molecular assays can be used to identify *Candida* pathogens directly from positive blood cultures (BCs) in a timely manner compared to standard methods using subcultures. In this study, the eazyplex^®^ *Candida* ID assay, which is based on loop-mediated amplification (LAMP) and is currently for research use only, was evaluated for the identification of the most common fungal species. A total of 190 BCs were analysed. Sensitivity and specificity were 93.88% and 99.26% for *C. albicans*, 89.13% and 100% for *Nakaseomyces glabratus* (*N. glabratus*), 100% and 100% for *Pichia kudravzevii* (*P. kudriavzevii*), 100% and 100% for *C. tropicalis,* and 100% and 99.44% for *C. parapsilosis*. Sample preparation took approximately 11 min and positive amplification results were obtained between 8.5 and 19 min. The eazyplex^®^ *Candida* ID LAMP assay is an easy-to-use diagnostic tool that can optimise the management of patients with candidemia.

## 1. Introduction

Invasive *Candida* disease, most commonly observed as candidemia or intra-abdominal infection, has a high mortality rate of up to 40% and affects vulnerable patients with underlying medical conditions such as cancer, diabetes, and cardiovascular disease [1]. Risk factors for mortality include immunosuppression, neutropenia, haemodialysis, mechanical ventilation, surgery, extensive use of medical devices, and use of broad-spectrum antibiotics [1,2]. Candidemia is often diagnosed late, which can lead to a worse clinical outcome in patients with more severe infections [2,3]. The early identification of *Candida* spp. in systemic infections is very important in order to start antifungal treatment as soon as possible [2,3]. The fungal antigens β-D-glucan and galactomannan are early biomarkers for monitoring high-risk patients, but they are not sufficient for a definitive diagnosis [2]. Although often criticised for having unsatisfactory detection rates and not being fast enough for timely diagnosis, blood cultures (BCs) remain the gold standard for candidemia detection of [4,5]. A detection of *Candida* spp. in BCs usually indicates a true infection, and BC processing can easily be integrated into a continuous daily workflow using automated incubator systems [5,6]. Non-culture-dependent PCR assays performed on blood samples may provide faster results but are not really suitable for continuous routine diagnostics because they are either too laborious or, in the case of fully automated test systems such as the T2Candida^®^ assay (T2 Biosystems, Lexington, MA, USA), very expensive [7,8]. Therefore, the development of diagnostic approaches for rapid fungal pathogens detection in candidemia and sepsis is a challenge to improving timely intervention with appropriate antifungal therapy.

The shorter the time to positivity of a BC, the higher the patient mortality rate [3]. Isolating yeast from positive BCs on agar plate subcultures followed by MALDI-TOF identification after several hours to a day delays diagnostic reporting. Performing MALDI-TOF identification directly from the BC bottle is an alternative strategy, but it increases the hands-on time for sample processing and shows poor performance for mixed infections [6,9]. In recent years, fully automated PCR tests have been developed for bacterial and fungal pathogen detection in positive BCs, namely the BioFire^®^ FilmArray^®^ (bioMérieux, Nürtingen, Germany) and ePlex^®^ (Roche Diagnostics, Penzberg, Germany). They have proven to be reliable rapid diagnostic tools, providing results in 1.5 to 3 h with minimal hands-on time, but with high additional consumable costs [6,10,11]. An alternative rapid BC testing approach is based on loop-mediated isothermal amplification (LAMP) assays. LAMP chemistry uses a robust *Bst* DNA polymerase that catalyses high-speed amplification without DNA purification from blood-containing samples. In this study, we evaluated the eazyplex^®^ *Candida* ID LAMP Research-Use-Only (RUO) assay (AmplexDiagnostics, Gars-Bahnhof, Germany) as a rapid diagnostic tool for the identification of common *Candida* species directly from positive BCs. The assay is supplied with ready-to-use lyophilised master mixes that can be stored at room temperature. It contains primers for *C. albicans, Nakaseomyces glabratus* (formerly *N. glabratus*), *Pichia kudravzevii* (formerly *P. kudriavzevii*), *C. tropicalis*, and *C. parapsilosis*, the most common species causing candidemia, and *C. auris* [12,13,14].

## 2. Materials and Methods

### 2.1. Candida Strains and Clinical BCs

The following reference strains purchased from LGC standards (Wesel, Germany) or obtained from the National Reference Center for Invasive Fungal Infections (NRZMyk), Leibniz Institute of Natural Product Research and Infection Biology-Hans Knöll Institute (Jena, Germany) were used to confirm the specificity of the eazyplex^®^ *Candida* ID (AmplexDiagnostics) primer sets: *C. albicans* DSMZ SC5314, *C. dubliniensis* ATCC 44508, *N. glabratus* ATCC 90030, *P. kudriavzevii* ATCC 90878, *C. tropicalis* ATCC 90874, *C. parapsilosis* ATCC 22019, *C. orthopsilosis* JMRC/STN01155, *C. metapsilosis* JMRC/STN00654. The *C. auris* strain HSM2/Ospedale San Martino, Genua, Italy, was obtained from AmplexDiagnostics. Clinical samples were BCs submitted to the clinical microbiology laboratory between July 2020 and September 2023 as part of routine patient care at the Jena University Hospital. 

### 2.2. Conventional BC Processing, Species Identification, and AST

Blood samples collected in BD BACTEC Plus aerobic/F and lytic/10anaerobic/F bottles (BD Diagnostics, Heidelberg, Germany) were incubated on a BACTEC FX instrument (BD Diagnostics). Positive BCs were aseptically sampled, Gram-stained, and routinely streaked onto Columbia sheep blood agar, chocolate agar, Drigalski lactose agar, and Schaedler KV agar (Thermo Fisher Scientific, Wesel, Germany) for overnight incubation at 37 °C. Once yeasts were identified by microscopy, a BC broth aliquot was additionally streaked onto CHROMagar^TM^ Candida plus (Mast Diagnostika, Reinfeld, Germany). Colonies were identified by Vitek MS (bioMérieux, Nürtingen, Germany). Antifungal susceptibility testing (AFST) was performed by determining minimal inhibitory concentrations (MICs) using the MICRONAUT-AM Antifungal Agents MIC microtiter system (Bruker Daltonics, Bremen, Germany; distributed by Sifin Diagnostics, Berlin, Germany). Breakpoints and epidemiological cut-off values (ECOFFs) were interpreted according to the criteria of the European Committee on Antimicrobial Susceptibility Testing (EUCAST; v10.0, https://www.eucast.org/astoffungi/clinicalbreakpointsforantifungals, accessed on 4 February 2020 and v4.0, https://www.eucast.org/fileadmin/src/media/PDFs/EUCAST_files/AFST/Clinical_breakpoints/EUCAST_BP_ECOFF_v_4.0.pdf, accessed on 14 August 2023). Due to the lack of *C. auris* infections in the hospital, simulated BC bottles were prepared. Colonies of a *C. auris* reference strain were suspended in tryptone soya broth (Thermo Fisher Scientific) to a 0.5 McFarland value and then diluted to 10^5^ CFU/mL. A 0.4 mL aliquot was added to 5 mL defibrinated sheep blood, inoculated into a BACTEC Plus Aerobic/F and lytic/10anaerobic/F bottle, and incubated as above. 

### 2.3. Eazyplex^®^ Candida ID LAMP Assay

The eazyplex^®^ *Candida* ID RUO assay is a ready-to-use test strip containing lyophilized master mixes with primers for *C. albicans*, *N. glabratus*, *P. kudriavzevii*, *C. tropicalis*, *C. parapsilosis*, and *C. auris* and an inhibition control with one specific primer set in each cap (Table 1). For LAMP testing, 25 μL of BC broth was added to 100 μL of Copan SL solution (phosphate-buffered saline containing dithiothreitol, Mast Diagnostica, Reinfeld, Germany) and vortexed. For control experiments with reference strains, a single colony was used instead of the BC broth. Then, 50 μL of this suspension was mixed with 50 μL of magnetic beads MA solution (AmplexDiagnostics) and boiled for 5 min. The tubes were placed in a magnetic rack (MagRack^TM^ 6; Cytiva, purchased from AmplexDiagnostics) and left for 1 min. Further, 25 μL of the clear solution was pipetted into 500 μL of resuspension and lysis fluid (RALF buffer, AmplexDiagnostics). Then, 25 μL of this mixture was pipetted into each well of the eazyplex^®^ test strip. Tests were run on a Genie HT machine (AmplexDiagnostics) at 65 °C for 30 min. Amplification was measured with real-time fluorescence detection using a DNA intercalating dye. Data interpretation was automatically performed using the integrated eazyReport^TM^ software (version v4.08). The results were reported as positive in real-time if the fluorescence level and the peak of the first derivative of the fluorescence curve exceeded thresholds of 10,000 and 0.025, respectively. The thresholds represent the default settings recommended by the manufacturer of the Genie HT instrument. 

### 2.4. Data Analysis

The eazyplex^®^ *Candida* ID assay’s performance was assessed by calculating sensitivity, specificity, and positive and negative predictive values (PPV and NPV) compared to Vitek MS, defined as the reference method. Cohen’s κ coefficient analysis was used to examine the agreement between the two diagnostic tests. Time to BC positivity and time to *Candida* species identification for the subculture and Vitek MS methods were calculated using times recorded in the laboratory information system. 

## 3. Results

Primer specificity was first verified using reference *Candida* strains (Table 2). All species strains included in this assay were correctly identified. In silico predicted cross reactions of the *C. parapsilosis* primers were confirmed for *C. orthopsilosis* and *C. metapsilosis*. Both species are cryptic species of the *C. parapsilosis* complex and the primers must therefore be defined as *C. parapsilosis* complex primers. The *C. albicans* primers targeting a mitochondrial gene sequence also gave a positive signal for *C. dubliniensis*, a closely related species. Due to the lack of mitochondrial genome data for *C. dubliniensis* in the NCBI database, the primer set specificity for species within the *C. albicans* complex could not be precisely defined. There was no cross reaction of any primer set with *Cryptococcus neoformans*.

**Figure 1:**
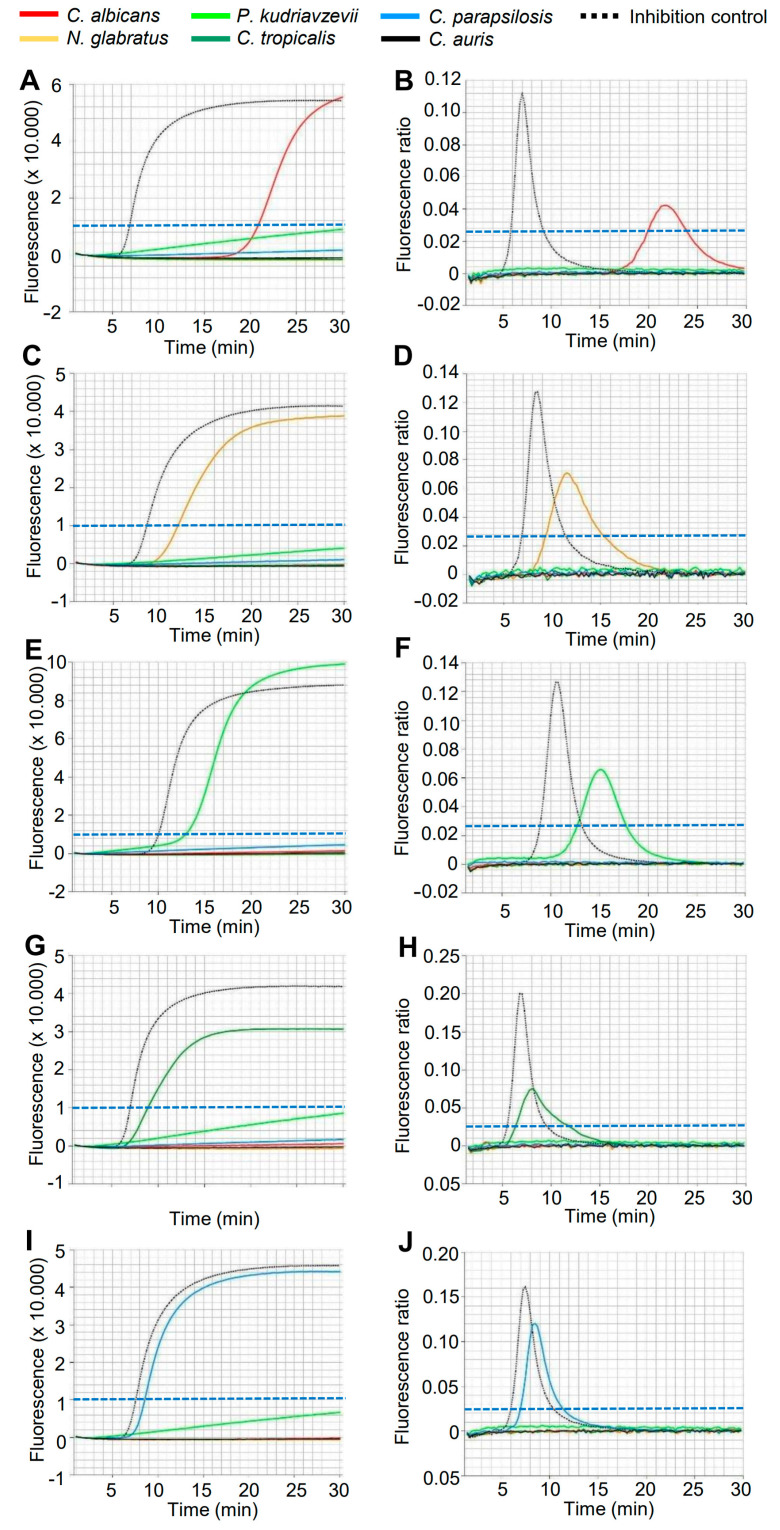
Detection of *Candida* species from positive BCs on the Genie HT instrument in less than 30 min. (**A**,**B**) *C. albicans*. (**C**,**D**) *N. glabratus*. (**E**,**F**) *P. kudriavzevii*. (**G**,**H**) *C. tropicalis*. (**I**,**J**) *C. parapsilosis*. The fluorescence level (**A**,**C**,**E**,**G**,**I**) and amplification rate (**B**,**D**,**F**,**H**,**J**) thresholds are marked with a broken blue line.

A total of 140 positive BCs showing yeast cells under the microscope and 50 negative BCs were analysed by LAMP; the results were compared with routine diagnostic species identification. The mean time to positivity of the BC bottles, defined as the time between the start of incubation and the positive signal, ranged from 15 to 71 h, depending on the species and bottle type (Table 3). MALDI-TOF MS species identification using subcultures took an additional 20 to 40 h in our routine workflow (Table 3). For the eazyplex^®^ assay, sample preparation took about 11 min (Table 3, legend). The time to result for the eazyplex^®^ *Candida* ID assay, defined by the threshold time of fluorescence intensity, ranged from about 9 min for *C. tropicalis* to 19 min for *C. albicans* (Table 3). Representative amplification curves of clinical BC samples are shown in Figure 1. As there was no clinical BC with *C. auris* growth, a spiked BC was examined and the identification of *C. auris* was confirmed (Table 3). Five samples had an invalid assay inhibition control and were excluded from the evaluation (3.57%).

The results for clinical BCs are summarised in Table 4. As expected, the most common species was *C. albicans*, with 49 cases. The eazyplex^®^ *Candida* ID missed three isolates. One positive result identified *C. dubliniensis* in subculture by MALDI-TOF, and the LAMP result was classified as false positive. Further, 41 out of 46 BCs with *N. glabratus* growth, the second most common species, were correctly identified by the eazyplex^®^ *Candida* ID, with no false positive results observed. Seven positive LAMP results for the *C. parapsilosis* complex were confirmed by MALDI-TOF as *C. parapsilosis* sensu stricto (n = 6), and one case of *C. orthopsilosis*. All *P. kudriavzevii* and *C. tropicalis* isolates were detected by LAMP, and no false positive results were observed. Additionally, 13 BCs that showed yeast cells after Gram staining but were negative in the eazyplex^®^ *Candida* ID revealed the following non-target species: *C. lusitaniae*, n = 6; *C. kefyr*, n = 4; *Trichosporon asahii*, n = 3, and *Saccharomyces cerevisiae*, n = 1. 

Overall, the eazyplex^®^ *Candida* ID assay demonstrated an acceptable direct BC testing accuracy in terms of sensitivity and specificity, compared to MALDI-TOF identification of subcultured colonies, as indicated by Cohen’s k values ≥ 0.92 (Table 4).

Mixed *Candida* infections were detected in five BC bottles. The eazyplex^®^ *Candida* ID results agreed with the subculture identification in three cases. In one case of a mixed infection of *C. albicans* and *N. glabratus,* LAMP detected only *C. albicans*. In another case, LAMP identified *C. albicans* and *C. parapsilosis*, but only *C. albicans* was detected in subculture. As no cross-reactions between the two species were predicted or observed, it is most likely that *C. paraspsilosis* was overlooked in the subculture. In BC cultures with mixed *Candida* spp. and bacteria infection, the fungal species was correctly identified when included as a target (7 out of 8 cases).

The AFST results did not show any unusual resistance patterns for any species. All MIC values for anidulafungin and micafungin were below the ECOFFs proposed by EUCAST. Fluconazole resistance was not observed in *C. albicans*, *C. tropicalis*, or *C. parapsilosis*. *N. glabratus* had a median MIC of 2 mg/L for fluconazole, and 8.3% of isolates were resistant.

## 4. Discussion

Delaying antifungal therapy in patients with candidemia is associated with increased mortality. Rapid *Candida* identification in blood samples is important to optimise patient treatment [6,15]. BCs remain the diagnostic reference method despite their suboptimal sensitivity, which can reach up to 50% [16]. Another critical issue is the time to result because *Candida* grows slower than the most common bacteria, resulting in a higher median time to positivity for BCs, and its identification from subcultures can also take longer [17]. Molecular testing systems can be used to rapidly identify *Candida* species directly from a BC bottle when it signals positive [6]. The results of this study show that a LAMP-based diagnostic assay can reliably identify the most common *Candida* species in less than 45 min including sample preparation, without the need for DNA extraction.

The question is whether species identification has a significant impact on antifungal treatment targeting before phenotypic AFST results are available. Echinocandins, such as caspofungin or anidulafungin, and azoles, such as fluconazole, are primarily used for the treatment of candidemia [15,18]. Echinocandins inhibit the synthesis of 1,3-β-glucan, and resistance can develop due to mutations in the hot spot region of the FKS genes encoding a subunit of the 1,3-β-glucan synthase [19]. Azoles inhibit the synthesis of cell wall-associated ergosterol. Resistance is mediated by mutations in the ERG-1 region of lanosterol 14-α-demethylase [19]. According to the guidelines of the European Society of Clinical Microbiology and Infectious Diseases (ESCMID) and the Infectious Diseases Society of America (IDSA), echinocandin use is recommended for initial candidemia treatment of due to intrinsic resistance or increased MIC values against fluconazole in some species, such as *P. kudriavzevii* and *N. glabratus*, and the association with a better outcome [20,21,22]. On the other hand, depending on geographic regions, an increase in isolates with reduced susceptibility against echinocandins has already been reported, such as for *C. parapsilosis* [23]. Therefore, fluconazole can be considered as an alternative drug for the treatment of *C. albicans*, *C. tropicalis,* and *C. parapsilosis* infections in haemodynamically stable patients with no recent azole exposure and without neutropenia [21]. Knowledge of the local antifungal susceptibility patterns of *Candida* species is important for eventual species-specific treatment [24]. In this context, it should be noted that phenotypic resistance testing also carries a risk of MIC value misinterpretation due to the heteroresistance phenomenon between subpopulations of a *Candida* species [25,26]. Therefore, the use of up to five different colonies for AFST in order not to miss resistant subpopulations is recommended [26].

The eazyplex^®^ *Candida* ID LAMP assay only includes the most common species targets as the panel is limited by the test strip format of eight wells for each specific primer mix. With the exception of *P. kudriavzevii*, all of these species are listed by the World Health Organization (WHO) as high- or medium-priority fungal pathogens due to the need for improved diagnostic tests and clinical outcomes [27]. Arguably, considering species with intrinsic or frequently acquired resistance to fluconazole and echinocandins may be most important for rapid BC testing. The most common pathogen is *C. albicans*, which typically has low antifungal resistance rates [12,28]. It is mainly responsible for invasive fungal infections in intensive care patients and is often associated with surgical interventions [24]. We observed a cross-reaction of the eazyplex^®^ primer set for *C. albicans* with *C. dubliniensis*. However, this will not have a direct clinical impact as *C. dubliniensis* is related to *C. albicans* and rarely develops resistance [29]. In this work, non-*C. albicans* species accounted for about half of the candidemia cases, in agreement with other recent studies [12,13]. Among these, *N. glabratus* was the most common. Formerly known as *C. glabrata*, this yeast has a haploid genome with strong plasticity, is characterised by elevated MICs against fluconazole compared to other species, and can develop resistance against both fluconazole and echinocandins after treatment [19,25]. *N. glabratus* forms a species complex with *N. nivariensis* and *N. bracarensis* [30]. Candidemia caused by *N. glabratus* often occurs in haematological and transplant patients with a history of antifungal treatment [2]. Some isolates of *N. glabratus* were not detected by the eazyplex^®^ assay. The primer set targets the ITS region of the rDNA, which has also been used to design PCR primers to differentiate species in the *N. glabratus* complex [30]. The eazyplex^®^ LAMP primers were selected to be specific for *N. glabratus* sensu stricto, but ITS point mutations may cause the failure to identify all strains. Unfortunately, isolates of *N. bracarensis* and *N. nivariensis* were not available to rule out cross-reactions within the species complex. *P. kudriavzevii* (formerly *C. krusei*) and *C. tropicalis* are species frequently isolated from patients with haematological malignancies [2,13]. While *P. kudriavzevii* has intrinsic fluconazole resistance, most *C. tropicalis* strains are susceptible to fluconazole; however, an increase in resistance rates has been reported [12,13]. All isolates of both species investigated in this study were correctly identified. *C. parapsilosis*, although reportedly associated with lower mortality, is a relevant fungal pathogen, showing potential to produce biofilms and persist in the hospital environment, posing a risk of intrahospital transmission [31]. Patients with haematological malignancies, recent surgery, intravascular devices, or parenteral nutrition are at risk of developing invasive infections [13]. Notably, *C. parapsilosis* may have higher MIC values for echinocandins due to the FKS1 polymorphism [24,31]. As shown here, the eazyplex^®^ assay identifies all species of the *C. parapsilosis* complex. *C. auris* is an emerging pathogen with the potential to cause outbreaks within hospitals because it is easily transmitted by direct contact and contaminated surfaces, unlike other species. [14]. Moreover, multidrug resistance and a high rate of relapse after treatment have been reported [32,33]. We found no clinical cases of *C. auris* infection in this study, but a simulated BC gave a correct result. High test specificity can be assumed as there were no false positive results for *C. auris* when other species were present in the BC.

Determining the regional spectrum of *Candida* species and their antifungal susceptibility patterns is critical for applying rapid BC testing in antifungal stewardship [34]. The failure to detect some *N. glabratus* isolates is a limitation of the eazyplex^®^ assay, as is the lack of primer sets for *C. lusitaniae* and *C. guillermondii*, which are inherently less susceptible to echinocandins due to FKS1 mutations [19]. To save time in diagnosis and timely start antifungal treatment, direct PCR testing of blood samples may be an alternative to BCs. However, few tests are commercially available and it is noteworthy that infections may sometimes also be missed, as reported for *N. glabratus* using the T2Candida^®^ assay [35]. Rapid BC testing is a promising add-on tool in candidemia diagnosis. The reliability of time-to-result PCR assays such as FilmArray^®^ and the ePlex^®^ has been demonstrated [10,11]. The advantages of the eazyplex LAMP assay are that results are available very quickly and reagent costs are lower, estimated at <EUR 30. Disadvantages include the need for some pipetting steps during sample preparation and the smaller number of species targets compared to the ePlex^®^ panel [11]. A limitation of this study is that it was not possible to statistically evaluate the assay’s identification of mixed fungal infections due to the small number of cases.

In conclusion, this study shows that eazyplex^®^ *Candida* ID is a rapid molecular BC assay that can be easily implemented in routine diagnostic workflows. Whether this assay is valuable for optimising patient management depends on the antimicrobial stewardship programme [12]. 

## Figures and Tables

**Table 1:** Target *Candida* genes used for LAMP primers of the *Candida* ID assay.

Assay Parameter	Gene	Gene Product	GenBank Accession No.	Predicted Cross-Reactions ^a^
*C. albicans*	*cox1*	Cytochromoxidase subunit 1	KC993188.1	None
*N. glabratus*	ITS	Internal transcribed spacer, rDNA	CR380947.2	None
*P. kudriavzevii*	*cox1*	Cytochromoxidase subunit 1	CP039616.1	None
*C. tropicalis*	*cox3*	Cytochromoxidase subunit 3	NC_022160.1	None
*C. parapsilosis*	*cox3*	Cytochromoxidase subunit 3	CP137564.1	*C. orthopsilosis*, *C. metapsilosis*, *C. theae*, *C. margitis*
*C. auris*	*nad5*	NADH dehydrogenase subunit 5	AP018713.1	None

^a^ Amplicon sequence homology > 95%.

**Table 2:** Identification of yeast reference strains using the eazyplex^®^ *Candida* ID assay to check for cross-reactivity of the primers.

Species ^a^	Eazyplex^®^ LAMP Results, Threshold Time (min)						
	*C. albicans*	*N. glabratus*	*P. kudriavzevii*	*C. tropicalis*	*C. parapsilosis*	*C. auris*	Inhibition Control
*C. albicans*	11. 92						8.8
*C. dubliniensis*	21.88						10.58
*N. glabratus*		10.2					12.27
*P. kudriavzevii*			10.58				9.01
*C. tropicalis*				6.78			9.93
*C. parapsilosis*					5.67		8.75
*C. orthopsilosis*					11.12		10.18
*C. metapsilosis*					9		8.97
*C. auris*						10.65	7.23
*C. neoformans*							12.2

^a^ For strain designation codes, see Section 2.

**Table 3:** Time to result of eazyplex^®^ *Candida* ID assay and subculture species identification from positive BCs by MALDI-TOF MS.

Species	n	Mean Time to Positivity of BCs (SD, h)		Eazyplex^®^ LAMP Mean Threshold Time (SD, min) ^a,b^	MALDI-TOF MS Species Identification Mean Time to Result from Subcultures (SD, h) ^c^
		Aerobic	Anaerobic		
*C. albicans*	47	38.6 (26.28)	28.45 (15.32)	18.93 (3.67)	22.55 (9.18)
*N. glabratus*	41	71.2 (24.39)	42.35 (25)	14.37 (5.15)	28.73 (10.02)
*P. kudriavzevii*	11	24.3 (12.06)	15.2 (8)	12.13 (2.15)	20.36 (12.6)
*C. tropicalis*	8	19.1 (10)	-	8.53 (1.95)	25.76 (8.38)
*C. parapsilosis* complex	8	28 (10.3)	32.3 (10.26)	11 (5.37)	39.7 (19.52)
*C. auris* ^d^	1	25.08	-	8	N.D.

^a^ Mean time of sample preparation (SD, min): 11.13 (0.4). ^b^ Mean threshold time (SD, min) of inhibition control: 9.47 (2.35). ^c^ Time from streaking BC aliquot on agar plates to identification result on following day (routine workflow). ^d^ Spiked BC.

**Table 4:** Performance data of the *Candida* ID assay for BCs.

LAMP Target	True Positive (n)	True Negative (n)	False Positive (n)	False Negative (n)	Sensitivity, % (CI ^a^)	Specificity, % (CI ^a^)	PPV ^b^, % (CI ^a^)	NPV ^c^, % (CI ^a^)	Cohen’s κ (CI ^a^)	Scale
*C. albicans*	46	135	1 ^d^	3	93.88 (83.13–98.72)	99.26 (95.94–99.98)	97.87 (86.70–99.69)	97.81 (93.72–99.26)	0.94 (0.89–1)	Almost perfect agreement
*N. glabratus*	41	139	0	5	89.13 (76.43–96.38)	100 (97.36–100)	100 (91.40–100)	96.50 (92.35–98.44)	0.92 (0.86–0.99)	Almost perfect agreement
*P. kudriavzevii*	11	174	0	0	100 (71.51–100)	100 (97.90–100)	100 (71.51–100)	100 (97.90–100)	1	Almost perfect agreement
*C. tropicalis*	8	177	0	0	100 (63.06–100)	100 (97.94–100)	100 (63.06–100)	100 (97.94–100)	1	Almost perfect agreement
*C. parapsilosis* complex	7	177	1 ^e^	0	100 (59.04–100)	99.44 (96.91–99.99)	87.5 (49.79–98.02)	100 (97.94–100)	0.93 (0.8-1)	Almost perfect agreement

^a^ CI, 95% confidence interval. ^b^ PPV, positive predictive value. ^c^ NPV, negative predictive value. ^d^ *C. dublinensis*., ^e^ unidentified in subculture.

## Data Availability

The dataset analysed in this study is available from the corresponding author upon reasonable request.
